# Severely exacerbated neuromyelitis optica rat model with extensive astrocytopathy by high affinity anti-aquaporin-4 monoclonal antibody

**DOI:** 10.1186/s40478-015-0259-2

**Published:** 2015-12-04

**Authors:** Kazuhiro Kurosawa, Tatsuro Misu, Yoshiki Takai, Douglas Kazutoshi Sato, Toshiyuki Takahashi, Yoichiro Abe, Hiroko Iwanari, Ryo Ogawa, Ichiro Nakashima, Kazuo Fujihara, Takao Hamakubo, Masato Yasui, Masashi Aoki

**Affiliations:** Department of Neurology, Tohoku University Graduate School of Medicine, Sendai, Japan; Department of Multiple Sclerosis Therapeutics, Tohoku University Graduate School of Medicine, Sendai, Japan; Department of Neurology, Faculty of Medicine, University of Sao Paulo, Sao Paulo, Brazil; Brain Institute, Pontifical Catholic University of Rio Grande do Sul (PUCRS), Porto Alegre, Brazil; Department of Neurology, Yonezawa National Hospital, Yamagata, Japan; Keio Advanced Research Center for Water Biology and Medicine, Keio University, Tokyo, Japan; Department of Pharmacology, School of Medicine, Keio University, Tokyo, Japan; Department of Quantitative Biology and Medicine, Research Center for Advanced Science and Technology, The University of Tokyo, Tokyo, Japan

**Keywords:** Animal model, Aquaporin 4, Astrocyte, Baculovirus display method, Neuromyelitis optica, Neutrophil

## Abstract

**Introduction:**

Neuromyelitis optica (NMO), an autoimmune astrocytopathic disease associated with anti-aquaporin-4 (AQP4) antibody, is characterized by extensive necrotic lesions preferentially involving the optic nerves and spinal cord. However, previous in-vivo experimental models injecting human anti-AQP4 antibodies only resulted in mild spinal cord lesions compared to NMO autopsied cases. Here, we investigated whether the formation of severe NMO-like lesions occurs in Lewis rats in the context of experimental autoimmune encephalomyelitis (EAE), intraperitoneally injecting incremental doses of purified human immunoglobulin-G from a NMO patient (hIgG_NMO_) or a high affinity anti-AQP4 monoclonal antibody (E5415A), recognizing extracellular domain of AQP4 made by baculovirus display method.

**Results:**

NMO-like lesions were observed in the spinal cord, brainstem, and optic chiasm of EAE-rats with injection of pathogenic IgG (hIgG_NMO_ and E5415A), but not in control EAE. Only in higher dose E5415A rats, there were acute and significantly severer clinical exacerbations (tetraparesis or moribund) compared with controls, within half day after the injection of pathogenic IgG. Loss of AQP4 was observed both in EAE rats receiving hIgG_NMO_ and E5415A in a dose dependent manner, but the ratio of AQP4 loss in spinal sections became significantly larger in those receiving high dose E5415A up to about 50 % than those receiving low-dose E5415A or hIgG_NMO_ less than 3 %. These lesions were also characterized by extensive loss of glial fibrillary acidic protein but relatively preserved myelin sheaths with perivascular deposition of IgG and C5b-9, which is compatible with post mortem NMO pathology. In high dose E5415A rats, massive neutrophil infiltration was observed especially at the lesion edge, and such lesions were highly vacuolated with partial demyelination and axonal damage. In contrast, such changes were absent in EAE rats receiving low-dose E5415A and hIgG_NMO_.

**Conclusions:**

In the present study, we established a severe experimental NMO rat model with highly clinical exacerbation and extensive tissue destructive lesions typically observed in NMO patients, which has not adequately been realized in in-vivo rodent models. Our data suggest that the pathogenic antibodies could induce immune mediated astrocytopathy with mobilized neutrophils, resulted in early lesion expansion of NMO lesion with vacuolation and other tissue damages. (350/350)

**Electronic supplementary material:**

The online version of this article (doi:10.1186/s40478-015-0259-2) contains supplementary material, which is available to authorized users.

## Introduction

Neuromyelitis optica (NMO), an autoimmune disease of the central nervous system (CNS), is clinically characterized by severe optic neuritis and longitudinally extensive transverse myelitis (LETM) [1]. About 70–90 % of the patients are seropositive for disease-specific autoantibodies [2, 3], such as NMO-IgG, which targets aquaporin-4 (AQP4) [4, 5], the water channel mainly localized to astrocytic foot processes [6]. NMO is characterized by a higher age of onset, female predominance [7], and greater autoimmune background than multiple sclerosis (MS) [8]. Clinical, MRI, and laboratory findings specific to NMO have been reported [7, 9–13]. The characteristic childhood-onset symptom of NMO is mainly optic neuritis, while that in elderly patients is myelitis [14], suggesting that the age of onset is associated with the localization of the lesion in NMO. The reason for the preferential involvement of optic neuritis and myelitis in NMO remains unclear. In addition to the optic nerve and spinal cord, there have been several reports on NMO lesions localized at circumventricular or periaqueductal areas, such as the area postrema [15] and hypothalamus [16], where AQP4 expression is enriched in the central nervous system [16]. However, the reason for the absence of NMO lesions in cerebral or cerebellar gray matter is still unknown. In a study of the cerebrospinal fluid (CSF), marked elevation of glial fibrillary acidic protein (GFAP) was evident in NMO, but not in MS, indicating that massive astrocyte lysis is a key to NMO pathology [9]. Moreover, pathological studies in autopsied NMO cases demonstrated extensive loss of astrocytic proteins, AQP4 and GFAP, especially in peri-vascular lesions with deposition of immunoglobulins and activated complement [17] and abundant infiltration of granulocytes and microglia [18]. In contrast, in these perivascular active lesions, myelin sheaths and axons are relatively preserved, suggesting a primary astrocytopathy [17]. We have reported that this vasculocentric AQP4 loss in the absence of myelin loss is a specific pathological feature in NMO, which has been reported both in early active lesions of autopsied NMO cases and a rodent model of NMO [17, 19]. Furthermore, in autopsied NMO cases, most of NMO cases tend to have extensive necrotic changes, and in the chronic phase in particular, the lesions show tissue softening and cavity formation [17, 18].

Experimental NMO models are needed for elucidating the underlying pathomechanisms and for testing candidate therapeutic drugs. Previous experimental NMO models have been useful to clarify the pathomechanisms of NMO, such as the pathogenicity of NMO-IgG in vitro [5] and that the NMO-IgG epitope is localized at the extracellular domains of membrane AQP4 [2, 5]. NMO-like lesion could not be reproduced by peripheral administration of only NMO-IgG, even in immature rodents with a leaky blood–brain barrier (BBB) or in BBB-permeabilized adult rodents [20]. These in vivo models have also clarified the participation of myelin-specific T cells in the development of these lesions [19, 21], and have reproduced astrocytopathic lesions upon loss of AQP4, while relatively preserving myelin sheaths [19, 22–25]. Moreover, they have identified IL-6 stimulating plasmablasts producing NMO-IgG [26] and have elucidated the role of IL-1β in the formation of NMO-like lesions [24]. It has become clear that a proinflammatory milieu, as well as NMO-IgG, is needed to generate an NMO-like pathology in rodent models [21]. Here, we presented the characteristics of in vivo experimental NMO models in Additional file 1 [19, 21–25, 27–32]. There are two major animal models of NMO. One is a NMO/Experimental autoimmune encephalomyelitis (EAE) model, involving intraperitoneal injection of NMO-IgG after the induction of EAE [19, 21–23, 27], while the other is a direct injection model (DI model), involving intracerebral, intrathecal or perichiasmal injection of NMO-IgG [25, 28–32]. The DI model can reproduce NMO-like lesions showing AQP4 loss and demyelination in the cerebral white matter, but the lesions are not always vasculocentric, are mostly localized around the injection site, and the injury caused by needle insertion poses a problem. In contrast, the NMO/EAE model can reproduce NMO-like lesions showing AQP4 loss mainly in spinal cord and this model is perhaps a more appropriate in vivo model, with CNS inflammation that induces movement of NMO-IgG across the BBB. Actually, in vivo experimental NMO rodent models have succeeded to partially reproduce NMO pathomechanism. For example, optic chiasma lesions were reproduced by continuous perichiasmal injection at the peri-injected sites in the DI model [31], but there is no adequate study for inducing an optic chiasma lesion in the NMO/EAE models. Furthermore, LETM lesions or lesions of the area postrema is one of the definitive features of NMO [1]; it has been reported that the size of the lesion, especially in the spinal cord, were quite small at each perivascular sites in most previous studies using pooled purified anti-AQP4 antibodies [19, 21, 22, 27], which is distinct from the diffuse extensive lesions observed in NMO. Those previous studies suggested that the reason for the difference in lesion localization and size in the DI and NMO/EAE models involves the reliability and accuracy of IgG access to the lesion site, and the quantity and volume of the IgG itself.

Therefore, in the present study, we hypothesized that *“large astrocytopathy comparable to NMO can develop and pathological features of NMO can be reproduced by injecting a massive dose of high-affinity anti-AQP4 monoclonal antibody (mAb) in the NMO/EAE model.”* To establish severe experimental NMO rat model closer to NMO pathomechanism, we used an AQP4-IgG derived from a baculovirus display method to generate a high-affinity and highly concentrated monoclonal IgG that specifically recognizes the extracellular domains of AQP4, and used it in the NMO/EAE model, after which we performed a detailed pathological examination in the acute phase.

## Materials and methods

### Animals

A total of 51 female Lewis rats were used in this study. Adult Lewis rats (LEW/CrlCrlj; 8–10-weeks-old, 140–180 g bodyweight-matched) were purchased from Charles River Lab (Yokohama, Japan). They were housed in the Institute for Animal Experimentation, Tohoku University Graduate School of Medicine, under standardized condtions. This study was approved by the ethical committee of the Tohoku University Graduate School of Medicine Committee on Animal Research (No.2015MdA-146).

### A NMO postmortem case

Here we present a double immunohistochemical study of AQP4 and complement C9neo in a case of typical NMO in Fig. 2a for better understanding. Her other sections were used in a previous study [33]. Briefly, she passed away during her last attack at 63 years old, having 5 episodes of bilateral optic neuritis and 6 histories of transverse myelitis. In pathology, marked inflammation consisting neutrophils and macrophages were observed with large necrotic centrally-located gray and white matter AQP4-lacked lesions with vasculocentric multiple isolated lesions especially localized in the periphery of spinal cord.

### Antibodies

- The purification of human IgG from sera in a healthy control (hIgG_cont_) and an NMO patient (hIgG_NMO_)
  Sera derived from a healthy person and an anti-AQP4-antibody-seropositive NMO patient were heated at 56.0 °C in a water bath for 30 min in order to inactivate the complement and preventing agglutination. The sera were clarified by centrifugation (4 °C , 3000 rpm, 10 min). In each sample, IgGs were captured by Protein A beads using a protein column system (rProtein A Sepharose Fast Flow, GE Healthcare, Tokyo, Japan), and were dialyzed through Cellu Sep T2 membranes (Membrane Filtration Products Inc, Texas, USA), and then finally concentrated to 1 mg/ml concentration. Antibody purifications were carried out after obtaining informed consent from the donors and from the ethical committee of Tohoku University Graduate School of Medicine (2014-1-652).
  A NMO patient of hIgG_NMO_ was a 68-year-old female who had a history of severe optic neuritis (left blindness) and myelitis, with 5-year disease duration. Her anti-AQP4 antibody titer was relatively high as 1:8,388,608 in our in-house cell-based assay, fulfilling the 2015 diagnostic criteria of NMO spectrum disorders [1]. She was negative for other autoantibodies, such as ANA, SS-A/Ro, and SS-B/La.
- Mouse monoclonal antibody against the extracellular domains of AQP4
  Monoclonal antibodies were established as previously reported [34, 35]. In brief, cDNA fragments encoding mouse AQP4 (mAQP4) M23 isoform in the E series or human AQP4 (hAQP4) M23 isoform in the D series [36] were inserted into a pBlueBac4.5 vector (Life Technologies, Carlsbad, CA, USA) to produce budded baculovirus (BV) expressing mAQP4 or hAQP4. To circumvent the immunological tolerance for mAQP4, we used AQP4-knockout mice (Acc. No. CDB0758K, ref. [37]) in E series as hosts, or used wild type mice in D series. Mice were immunized intraperitoneally with a phosphate-buffered saline solution containing BV expressing mAQP4 M23 or hAQP4 M23 isoforms and pertussis toxin. Flow cytometry and an ELISA (using CHO cells stably expressing mAQP4 M1 and M23, and human AQP4 (hAQP4) M1 and M23 isoforms) were used to screen the affinity of the antibodies for AQP4; we then chose two clones (E5415A and D15107) for use in rat NMO models, because these antibodies showed the highest affinity for mouse M1 and M23 (E5415A) and human M1 and M23 (D15017; data not shown), as previously reported [38].

### Cell-based affinity assay to assess binding of NMO-IgGs to rat AQP4

To perform a cell-based assay (CBA) for estimating binding affinities of all antibodies used in this study for rat AQP4, we established a CHO-cell clone expressing rat AQP4 M23. A cDNA encoding rat AQP4 M23 was cloned from total RNA extracted from rat cerebella by reverse transcription polymerase chain reaction, using primers 5′-GCTAGCATCATGGTGGCTTTCAAAGGAGTCTGGAC-3′ and 5′-CCGCGGTCATACAGAAGATAATACCTCTCCAGACG-3′. After sequencing, the cDNA was inserted into the *Nhe*I and the *Sac*II sites of a pIRES2-EGFP vector (Clontech Laboratories, Mountain View, CA, USA), in which the unique *Afl*II site had been changed to an *Eco*RI site by linker ligation. For establishment of CHO-cell clones stably expressing rat AQP4, the vector was linearized with *Eco*RI before transfection. Then, the linearized vector was transfected into CHO cells seeded onto 3.5-cm dishes at a density of 2 × 10^5^ cells/dish using Lipofectamine Plus reagents (Life Technologies, Carlsbad, CA, USA). Two days after transfection, cells were trypsinized and reseeded onto ten 10-cm dishes in medium containing G418 (500 μg/ml, Nacalai Tesque, Inc., Kyoto, Japan). Approximately 10 days after selection with G418, several colonies positive for fluorescence of EGFP were picked. After amplification and confirmation of AQP4 expression by western blotting, several single-cell clones were obtained by limiting dilution.

In stably cultured slides of the transfected cells, these cells were exposed to serially diluted IgG, including anti-AQP4 antibodies (4 °C temperature, overnight), followed by Alexa Fluor 568 or 594 IgG as the secondary antibody (room temperature, 1 h). A membrane-fluorescent pattern, at the rims of CHO cells, was considered to indicate the affinity of the anti-AQP4 antibody. All three NMO-IgGs, such as E5415A, D15107, and hIgG_NMO_ showed positive staining, but their affinity for rat AQP4 was markedly different in a double-dilution method, started at 1 mg/ml. Among the antibodies tested, E5415A showed the highest affinity for rat AQP4 (Additional file 2). Therefore, we selected E5415A as the highest affinity anti-AQP4 monoclonal antibody (mAb), and used it in our subsequent experiments. The IgG subtype of E5415A is IgG2a, with an ability to activate complement, as does the IgG1 subtype in human.

### NMO/EAE experiment—an experimental NMO rat model

- EAE induction
  Fifty-one Lewis rats, divided into the following groups: normal rats injected with hIgG_cont_ (*n* = 2), normal rats injected with hIgG_NMO_ (*n* = 2), normal rats with E5415A (*n* = 2), EAE not injected with antibody (*n* = 8), EAE with injection of 20 mg hIgG_cont_ (*n* = 7), 2 mg hIgG_NMO_ (*n* = 2), 20 mg hIgG_NMO_ (*n* = 6), 40 mg hIgG_NMO_ (*n* = 5), 80 mg hIgG_NMO_ (*n* = 1), 0.01 mg E5415A (*n* = 4), 0.1 mg E5415A (*n* = 5), or 1 mg E5415A (*n* = 7), were used in the present study. Initially, these rats were immunized with an encephalitogenic mixture containing guinea pig brain myelin basic protein (MBP; Sigma-Aldrich, Tokyo, Japan) in complete Freund’s adjuvant (CFA; Chondrex Inc, Redmond, WA, USA), which stimulates the disruption of the BBB and mobilizes activated T cells in the CNS to promote a proinflammatory milieu. Each animal received a single subcutaneous injection of 200 μl of an emulsified solution including 1 mg/ml MBP in PBS and CFA containing 1 mg/ml heat-killed H37Ra *Mycobacterium tuberculosis*.
- Intraperitoneal injection of IgGs
  About 2 weeks after the injection of the emulsion, ascending paresis (starting with a flaccid tail, followed by hindlimb paresis) developed in most rats (42/45, 93.3 %). Three rats did not show any symptoms and were excluded from this study; these included two in the EAE without injection group, and one in the relatively low-dose 0.01 mg E5415A group. At the time of clinical onset, we administered an intraperitoneal (IP) injection of IgG without complement. In the hIgG groups, we injected 20 mg hIgG_cont_ into Lewis rats (*n* = 7) as the normal control, and we injected different doses of hIgG_NMO_ into the test rats: relatively low-dose 2 mg (*n* = 2) or 20 mg (*n* = 6), and relatively high-dose 40 mg (*n* = 5) or 80 mg (*n* = 1). In the mIgG_NMO_ groups, we also injected different doses of E5415A into the Lewis rats: relatively low-dose 0.01 mg (*n* = 3), and relatively high-dose 0.1 mg (*n* = 5) or 1 mg (*n* = 7). In the 0.01 mg E5415A group, a rat was removed because no clinical exacerbation was noted. In the 1 mg E5415A group, two of the seven rats died, and these data were removed from the statistical analysis. The difference of the injection volume between hIgG_NMO_ and E5415A must be due to the affinity of each antibody against rat astrocytes (Supplement 2). We studied the blood kinetics of IP-injected NMO-IgG by antibody titration using an ELISA and a cell-based assay. This demonstrated that 12–24 h were required to attain a maximum blood concentration of NMO-IgG (Additional file 3).
- Clinical evaluation
  The bodyweight was measured daily in all rats and clinical disability scores were measured as follows. 0: No symptoms, 1: Flaccid tail, 2: Hindlimb paresis with gait abnormality, 3: Hindlimb plegia, complete dragging of the hindlimb, 4: Forelimb paresis, 5: Forelimb plegia or moribund (continuous hypopnea or bradycardia), 6: Dead.
- Immunohistochemistry
  Two days after the final injection of IgG, all the CNS tissues, including the brain, brain stem, optic nerves, and spinal cords were dissected from the rats, fixed for another 24 h in 4 % of paraformaldehyde (PFA), and embedded in paraffin according to standard procedures. Then, 4-μm-thick paraffin sections were cut and mounted serially onto numbered slides so that the distribution of molecules, such as AQP4 and GFAP, could be compared in adjacent serial sections. We stained all sections with hematoxylin and eosin (HE) and Klüver-Barrera (KB) staining. We used the avidin-biotinylated enzyme complex (ABC; Vectastain, Vector, CA, USA) or EnVision (Dako, Carpinteria, CA, USA). Briefly, at first, the paraffin sections on slides were immersed in xylene for 5 min three times, and then they were immersed in 100 % ethanol, 95 % ethanol, and then in 90 % ethanol for 5 min. After washing with distilled water, we washed the slides three times with PBS. Non-specific binding was blocked with 10 % goat serum or 10 % rabbit serum for 15 min at room temperature, and the slides were covered with a solution containing primary antibodies, and incubated for 1–24 h at the appropriate temperature. The slides were washed with PBS and incubated with PBS containing 30 % methanol and 1 ml of 30 % H_2_O_2_ for 20 min followed by three washes with PBS. The primary antibody was omitted in the control study. Then, secondary antibodies were applied and the slides incubated for 40 min to 1 h at room temperature, according to the manufacturer’s protocols. For staining, we used diaminobenzidine hydrochloride (DAB; brown) for the horseradish peroxidase (HRP) system, and fuchsin (Dako, Carpinteria, CA, USA) or Vector blue (Vector, Burlingame, CA) for the alkaline phosphatase (AP) system. Selected sections were counterstained with a filtered solution of hematoxylin (blue), methyl green (Vector, Burlingame, CA; green), or fast nuclear red (Vector, Burlingame, CA; red). In the present study, we used several primary antibodies: AQP4 (1:200; Santa Cruz, Texas, USA), EAAT2 (1:200; Abcam, Tokyo, Japan), GFAP (1:500; Proteintech, Chicago, USA), Iba-1 (1:200; Abcam, Tokyo, Japan), anti-mouse IgG Fc fragment specific (1:2000; Thermo Scientific, St. Louis, MO, USA), anti-rat C5b-9 (1:500; Hycult Biotech, Uden, Netherlands), MBP (1:500, Dako, Glostrup, Denmark), MAG (1:50; Sigma-Aldrich, St. Louis, MO, USA), and NF (1:1000; Calbiochem, San Diego, CA, USA).

### Statistical analysis

We compared the measurements in samples by the Mann–Whitney *U* test and 2-tailed p-values < 0.05 were considered significant. Results of measurements are shown in median and interquartile range—the top value represents the upper 25 % percentile, and the bottom value represents the lower 25 % percentile, unless otherwise indicated. Significance is indicated as *p < 0.05, **p < 0.005, ***p < 0.0005, and ****p < 0.00005.

## Results

### Clinical exacerbation of NMO/EAE models with E5415A and hIgG_NMO_ in dose-dependent manner

**Fig. 1:**
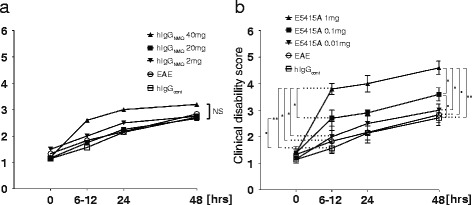
Clinically intensive exacerbation in the E5415A model. No significant (NS) clinical exacerbation occurred in the hIgG_NMO_ model (**a**). In contrast, acute and severe exacerbation was observed in rats receiving 0.1–1 mg E5415A, in an IgG-dose-dependent manner (**b**). Within 48 h after the IgG injection, the clinical disability score in the 1 mg E5415A models was statistically significant compared with those in the 0.1 mg (*p* = 0.0413), 0.01 mg (*p* = 0.0272), normal EAE (*p* = 0.0101), and hIgG_cont_ groups (*p* = 0.0038). In addition, the score in the 0.1 mg E5415A rats was also significantly higher than that in the hIgG_cont_ rats (*p* = 0.0280). These changes strikingly occurred at 6–12 h after the NMO-IgG injection. Within 6–12 h, the disability score in the 1 mg E5415A model was statistically significantly different to those in the 0.1 mg (*p* = 0.0209), 0.01 mg (*p* = 0.0376), normal EAE (*p* = 0.0160), and hIgG_cont_ groups (*p* = 0.0057), and the score in the 0.1 mg group was significantly higher than that in the hIgG_cont_ group (*p* = 0.0284)

In the high-dose E5415A groups (0.1–1 mg), clinical exacerbation was readily observed in an NMO-IgG dose dependent manner. The clinical course in this experiment is shown in Fig. 1. Ascending paresis developed in all EAE groups, however, the symptoms were temporary, and relatively mild, with flaccid tail (in 6/6 rats) and hindlimb paresis (4/6) being observed, but tetraparesis (1/6) was rare in EAE groups without antibody injection. The clinical exacerbation was not significantly different in the hIgG_cont_, hIgG_NMO_, and 0.01 mg E5415A groups (Fig. 1a)

In contrast, the clinical disability score in 1 mg E5415A models were statistically significant compared with those in 0.1 mg (*p* = 0.0413), 0.01 mg (*p* = 0.0272), normal EAE (*p* = 0.0101), and hIgG_cont_ model (*p* = 0.0038).

In addition, the score in 0.1 mg E5415A is also significantly higher than in hIgG_cont_ (*p* = 0.0280). The degree of clinical manifestations is as follows. At 48 h after IgG injection, the tetraparesis was found mostly in the groups receiving the higher amounts of E5415A; three of five rats in the 0.1 mg group, and all (5/5) rats in the 1 mg group. Two of five rats in the 1 mg group were tetraplegic or moribund, with continuous hypopnea and bradycardia. Two of seven rats in the 1 mg group were dead at this time-point, and were excluded from this analysis because of poor pathological examination. Interestingly, such intensive clinical exacerbation was marked at the hyper-acute phase ranging from 6 h to 12 h after E5415A injection (Fig. 1b), which coincided with a notable elevation of the E5415A concentration in the blood (Additional file 3).

In rats injecting E5415A or hIgG_NMO_ without immunization, there is no clinical exacerbation and we could not observe asymptomatic lesion with loss of AQP4 in the obex and area postrema in the present study.

### Lesion volume in the NMO/EAE model with dose dependent manner

**Fig. 2:**
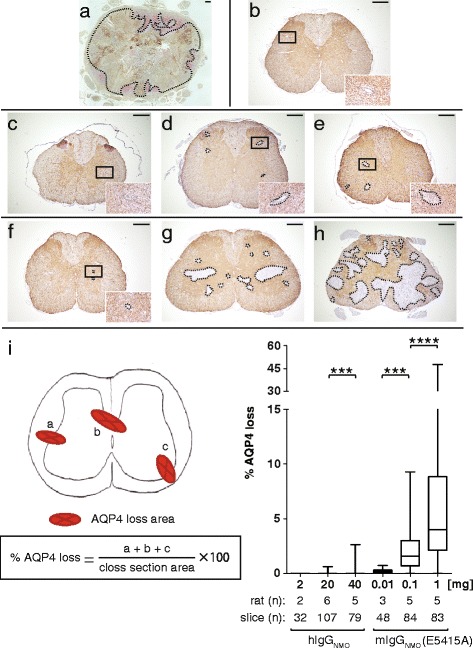
Loss of AQP4 in spinal cord legions occurs in a dose-dependent manner. The comparison of lesion size in response to doses of anti-AQP4 antibody is shown. Initial photograph (**a**) is a typical case of NMO patient for better understanding, showing extensive loss of AQP4 in the entire spinal cord. There are multiple rosette-like depositions of complement C9neo especially evident in the gray matter and perivascular areas of white matter, where vasculocentric loss of AQP4 is relatively enriched (**a**), especially in the gray matter and perivascular polarized expression of normal AQP4 staining (**b**). In hIgG_NMO_ rats (**c**−**e**) and in E5415A rats (**f**−**h**), loss of AQP4 was observed particularly at the corticomedullary junctions. The lesions gradually enlarged in a hIgG_NMO_ dose-dependent manner, from 2 mg (**c**), 20 mg (**d**), to 40 mg (**e**). Similarly, the lesion enlarged in E5415A rats, from 0.01 mg (**f**), 0.1 mg (**g**), to 1 mg (**h**), in a dose-dependent manner. The region showing AQP4 loss in the 0.1 mg and 1 mg E5415A groups (**g**−**h**) was markedly greater than that observed in the hIgG_NMO_ group (**c**−**e**). The maximum size of the region of AQP4 loss in the 1 mg E5415A group (**h**) was comparable to that seen in an NMO postmortem case (**a**). The percentage of AQP4 loss in spinal cord sections was calculated in each group (**i**). The ratio in the higher IgG group of hIgG_NMO_ or E5415A was significantly higher than that in the lower IgG groups. Scale bar = 300 μm

In autopsies of typical NMO cases, large lesions with astrocytopathy are observed, with predominant involvement of gray matter and diffuse expansion over areas containing multi-vessels, and necrotic changes are occasionally seen (Fig. 2a). In the hIgG_cont_ group after EAE induction, there was no AQP4 loss (Fig. 2b). In contrast, injection of hIgG_NMO_ could induce some small multi-vessel lesions in a dose-dependent manner (Fig. 2c−e). However, the size of these lesions was markedly smaller than those seen in the typical NMO-postmortem cases (Fig. 2a). In an experiment of a single 80 mg hIgG_NMO_ injection, we observed diffuse lesions comparable in size to the lesions in the 0.1 mg E5415A group (data not shown), with neutrophil infiltration.

Using the high-affinity anti-AQP4 mAb E5415A, a dose-dependent loss of AQP4 was observed particularly in the peri-vascular areas (Fig. 2f−h) to a much greater extent than the AQP4 loss observed in the hIgG_NMO_ model (Fig. 2i). The maximum size of these lesions exceeded half the size of the entire cross section of the spinal cord (Fig. 2h). Some lesions of myelitis extended longitudinally and transversely (LETM) as usually observed in NMO cases. We examined 1 mg D15107 NMO/EAE model, but lesions are quite smaller than the same dose of E5415A model, with few neutrophil infiltrations (data not shown).

### In vivo model of primary astrocytopathy

In the MBP-EAE model without antibody injection, lymphocyte and microglia infiltration was clearly observed, particularly in the white matter in the peri-vascular and subpial areas, mostly in the form of perivascular cuffing. However, AQP4 loss, demyelination, axonal injury, and neutrophil infiltration were not observed in the MBP-EAE model.

**Fig. 3:**
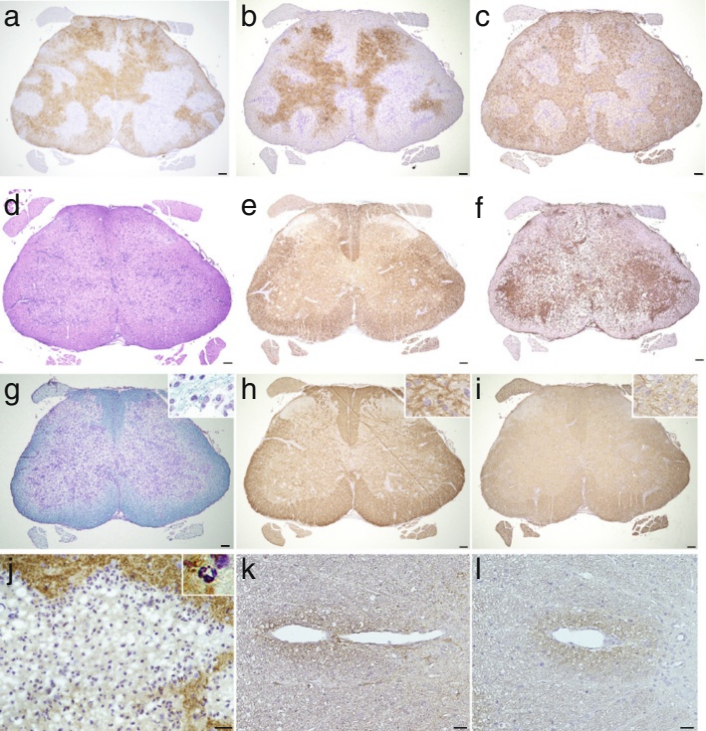
IgG and complement mediates extensive astrocytopathy in E5415A model. Extensive loss of AQP4 (**a**), EAAT2 (**b**), and GFAP (**c**) were observed particularly in the inflamed peri-vascular areas (**d**) positive for Iba1 (**f**); in contrast, myelin fibers and neurofilament (NF) staining was relatively preserved (**e**), suggesting a primary astrocytopathy. (**g**) Myelin pallor was particularly noticeable in the gray matter upon KB staining. (**h**) MBP staining was virtually intact, but KB and MAG staining was weak, particularly in the cortico-medullary junctions (**g−i**), and myelin debris could be observed mildly around inflamed sites with KB, MBP, and MAG staining (upper right corner: **g**−**i**). These findings suggest insidious early demyelination. (**j**) Abundant neutrophils were present especially at the lesion border of AQP4-lacking lesions with numerous tissue vacuolations. (**k**−**l**) Anti-mouse IgG (**k**) and anti-rat C5b-9 (**l**) deposition were observed in the peri-vascular areas. Scale bar = 100 μm (**a**−**l**), 40 μm (**j**), and 20 μm (**k**−**l**)

In contrast, in the 0.01–1 mg E5415A group, loss of AQP4, EAAT2, and GFAP was marked in the Iba1-positive perivascular areas, especially in 1 mg E5415A (Fig. 3a−f), in which multiple perivascular localization of lesions have previously been reported in autopsied NMO cases and in some experimental NMO models [17, 19]. In contrast, the staining for KB, MBP, MAG, and NF remained relatively preserved (Fig. 3e, g−i), suggesting a primary astrocytopathy. Neutrophils were often observed in lesion sites with tissue vacuolation (Fig. 3j).

These perivascular lesions were often stained by anti-mouse IgG and anti-rat C5b-9 Ab, suggesting the involvement of antibody- and complement-dependent cytotoxicity in this model (Fig. 3k, l). The IgG and complement deposition formed a rosette-like pattern or a rim-pattern, as previously reported in NMO [18].

The staining of MBP was mostly intact (Fig. 3h). In contrast, MAG staining was generally pale and large amounts of small myelin debris were observed around the inflamed vessels (Fig. 3i), suggesting that the lesions were caused by dying-back of oligodendrocytes, termed “distal oligodendrocytopathy” [39].

### Massive neutrophil infiltration precedes tissue vacuolation

**Fig. 4:**
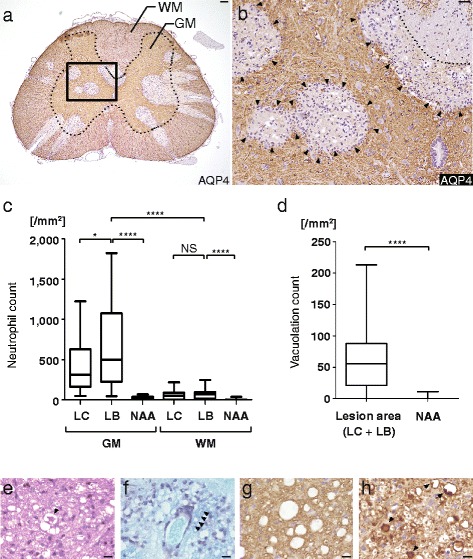
Pathology of neutrophils and vacuolations in the E5415A model. Neutrophils and tissue vacuolation was strongly observed in the E5415A groups (0.1–1 mg), but never observed in the normal EAE, EAE with hIgG_cont_, hIgG_NMO_, and E5415A (0.01 mg) groups. Lesions were typically observed in a linear arrangement along radiating vessels and were diffusely present in the gray matter (**a**). The localization of neutrophils in the E5415A model (1 mg) is shown (**b**). The neutrophils were more abundantly present in the gray matter (GM) than in the white matter (WM) at spinal cord lesions (**a**, **c**), particularly at the lesion border (LB) (**b**). In the gray matter, the counts of neutrophils were significantly higher in the LB, than in the lesion core (LC) and normal appearance area (NAA) (**c**). Furthermore, there was a marked difference in tissue vacuolation in areas lacking AQP4 expression compared with NAA, statistically significant (**d**), particularly in the LC (**b**). The edges of these vacuoles were sometimes stained upon KB (**f**), MBP (**g**), or NF staining (**h**). KB staining was weak around the peri-vascular lesions, where foam-like vacuoles were found along with myelin debris (arrowhead) (**f**). Furthermore, some vacuoles included eosinophilic (**e**, arrowhead) and/or densely NF-positive distorted structures (**h**, arrowhead), suggesting degenerated and/or swollen fibers. These findings suggested the early active lesions with intra-myelinic edema and axonal injury. Scale bar = 100 μm (**a**), 40 μm (**b**), and 10 μm (**e**−**h**)

The infiltration of neutrophils was not observed in the EAE without injection, EAE with hIgG_cont_, hIgG_NMO_, and E5415A (0.01 mg) groups, but was present in the E5415A-injected groups (0.1–1 mg), i.e. in 52 of 84 spinal cord axial sections (62 %) in five rats in the 0.1 mg group and 83 out of 83 sections (100 %) in five rats in the 1 mg group. In these sections, more than 500 neutrophils/mm^2^ were observed in 10.7 % sections in the 0.1 mg and 43.4 % in the 1 mg groups. Many more neutrophils were observed in the gray matter than in the white matter in the spinal cord lesions at 48 h after the injection of NMO-IgG (Fig. 4a). When we examined 30 spinal cord sections well observed neutrophil in 1 mg E5415A model, in the gray matter, neutrophil counts were significantly higher in the lesion border (LB) than in the lesion core (LC) and normal appearance area (NAA) (Fig. 4c). Furthermore, there was a marked difference in tissue vacuolation in areas lacking AQP4 expression (Fig. 4d), especially in the LC (Fig. 4b). Given that NMO shows a vasculocentric pathology that spreads outward, these findings show that the neutrophils tend to be localized at the lesion edge where there is high AQP4 expression.

In the lesions in the E5415A injection (0.1–1 mg) groups, tissue vacuolation was diffusely present in areas lacking AQP4 and GFAP, along with infiltration of numerous neutrophils. In contrast, such tissue destructive changes were not observed in the hIgG_NMO_ or E5415A (0.01 mg) groups. The morphology of the vacuoles was spherical or oval, but vacuoles frequently fused with each other and took on a larger, irregular shape, particularly in the lesion area. The edge of these vacuoles was sometimes stained with KB (Fig. 4f), MBP (Fig. 4g), or NF (Fig. 4h). KB staining was weak around the peri-vascular lesions, where bubble-like vacuoles were found along with myelin debris; in contrast, neutrophils were seen at the lesion border (Fig. 4f). Furthermore, only in E5415A (1 mg), some vacuoles included eosinophilic fine structures with or without densely NF-positive debris, suggesting axonal swelling and damage (Fig. 4e, h). In 83 lesion-expanded sections of 5 rats with E5415A (1 mg) injection, which have no diffuse loss of neurofilament staining, the morphology of axon is not intact in 23 lesions (27.7 %) and is relatively severely damaged such as axonal swelling or debris in 8 lesions (9.6 %), suggesting focal or central core damage and the existence of diverse pathology. In contrast there is no marked abnormality in rat groups of E5415A (0.1 mg, 0.01 mg) and hIgG_NMO_. These findings suggest an early active lesion with intra-myelin edema and axonal injury. Vacuolation was diffusely present in lesion areas lacking AQP4 and GFAP (Fig. 4d); it was observed only at the peri-vascular areas in early lesions (Fig. 4b) with disintegrated GFAP-positive foot processes, suggesting that the lesions of this model are of vasculocentric origin, with BBB disruption, followed by secondary extension of neutrophil infiltration and tissue vacuolation.

### Lesion localization in NMO/EAE models

**Fig. 5:**
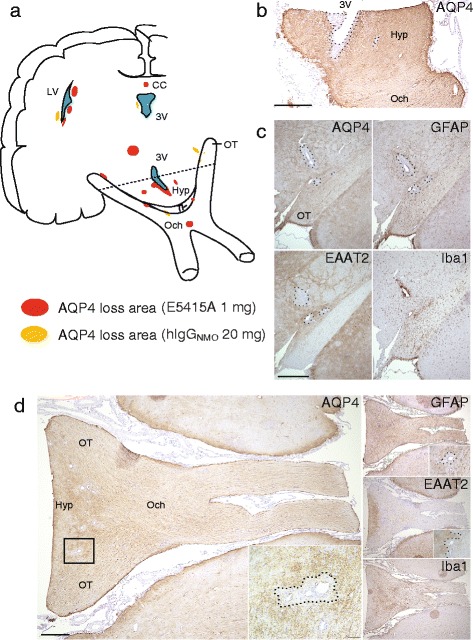
Pathology of optic neuritis and brain lesions. The localization of AQP4 loss in the optic nerve, peri-ventricles, and hypothalamus in the 1 mg E5415A injection (red in schema) and hIgG_NMO_ (yellow in schema) (**a**). Peri-3^rd^ ventricle lesions were bilaterally and diffusely extended along the ventricular wall (**b**). Optic tract lesion and optic chiasma lesion in a 20 mg hIgG_NMO_ rat is shown (**c**−**d**). AQP4, EAAT2, and GFAP loss were observed in Iba1-positive peri-vascular areas. Optic chiasma lesions were located in the perivascular areas of the anatomical border between the optic chiasma and hypothalamus (**d**). Scale bar = 300 μm

We detected some NMO-like lesions in the optic chiasma (Och), optic tract (OT), hypothalamus (Hyp), peri-3^rd^ ventricles (3 V), peri-lateral ventricles (LV), and corpus callosum (CC) at the optic nerve and brain in the NMO-EAE models. Fig. 5 shows the schema and the pathology of an optic nerve axial section and brain coronal section in these rats. As shown in Fig. 5a and d, Och and OT embraced the Hyp in the axial sections. The NMO-like lesions were observed at the perivascular areas of the OT (Fig. 5c) and Och, particularly in the border zone between the Och-OT and the Hyp (Fig. 5d). Throughout the lesions, the loss of AQP4, EAAT2, and GFAP could be observed in the Iba1-positive perivascular areas, surrounded by AQP4- and GFAP-positive reactive astrocytes. Furthermore, periventricular lesions were clearly observed in the NMO/EAE models and Hyp lesions adjacent to 3 V are shown in Fig. 5b.

**Fig. 6:**
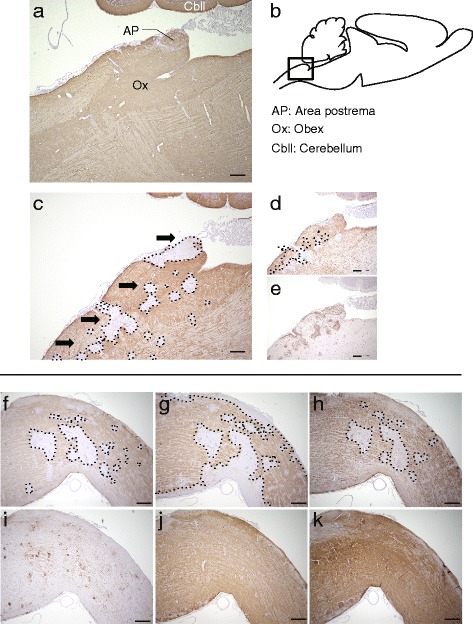
Pathology of the area postrema and medulla oblongata. Immunostaining of AQP4 in a sagittal section of the brain of a rat that had received an injection of hIgG_cont_ (**a**) and a schema of the sagittal rat brainstem (**b**). In the 1 mg E5415A group, there were several AQP4-lacking lesions with a longitudinally extended moniliform appearance, from the obex and the area postrema to the upper cervical cord (**c**). Compared with injection of hIgG_cont_ (**a**), the marked loss of AQP4 was observed in the area postrema and the obex (**b**). Immunostaining for GFAP was weak at the area postrema and in multiple perivascular lesions (**d**) around Iba1-positive vessels (**e**). Furthermore, medulla oblongata lesions demonstrated typical NMO pathology, including extensive loss of AQP4 (**f**), EAAT2 (**g**), and GFAP (**h**) at Iba1-positive perivascular regions (**i**), but the myelin sheath (**j**) and neurofilament (**k**) were relatively preserved. Scale bar = 300 μm

In the brain stem, we found lesions in the area postrema (AP) and medulla oblongata in the 1 mg E5415A group. Immunostaining for AQP4 in the sagittal section of a hIgG_cont_ rat is shown in Fig. 6a, and a schema of the sagittal rat brainstem is shown in Fig. 6b. At the AP in the 1 mg E5415A group, AQP4 loss was obvious and GFAP staining was weak (Fig. 6c, d). In contrast, in Iba1-positive perivascular lesions around the AP, GFAP and AQP4 loss was clear (Fig. 6c−e). Interestingly, a row of lesions was seen along the obex (Fig. 6c−e). Similar to spinal cord lesions [15], the medulla oblongata lesion showed features of extensive primary astrocytopathy, in which large areas showing loss of AQP4, EAAT2, and GFAP were observed, but MBP and NF expression was relatively preserved (Fig. 6f−k).

**Fig. 7:**
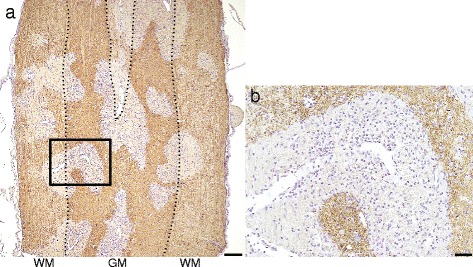
Pathology of longitudinally extensive transvers myelitis-like lesions. Sagittal section of the spinal cord lesion in a 1 mg E5415A rat is shown (**a**) Multiple perivascular lesions, which were diffusely extended, coalesced with each to form lesions similar to longitudinally extensive transverse myelitis (LETM) lesions. Most of the infiltrating cells were polymorphonuclear leukocytes, and were markedly more present in the gray matter than in the white matter (**b**). These findings suggested that the mechanism underlying LETM is based on fusion of individual vasculocentric astrocytopathy lesions. Scale bar = 300 μm (Fig. 7a) and 40 μm (Fig. 7b)

We examined the pathology of sagittal sections in the spinal cord in the 1 mg E5415A group. Multiple perivascular regions with AQP4 loss coalesced to form longitudinally extensive spinal cord lesions, suggesting an LETM-like lesion (Fig. 7a). Most of the infiltrating cells were granulocytes (Fig. 7b), and the density of the infiltrated cells was more marked in the gray matter than in the white matter, as previously mentioned. These findings suggest that LETM formation is based on the fusion of neighboring vasculocentric astrocytopathic lesions as previously reported [33].

## Discussion

Several in-vivo NMO models, previously reported, were insufficient to reproduce the diffuse and large edematous lesions with astrocytopathy and necrotic tissue damage typically seen in NMO patients. Several reasons may underlie, but one possibility is due to the gap of specific epitope of human anti-AQP4 antibody and its affinity against rodent AQP4 because of different species. The other is the possible existence of diverse mechanisms influenced on the tissue damage observed in NMO such as antibody-dominant (ADCC) and complement-dominant cytotoxic (CDC) tissue damage [40, 41], that knockdown of complement inhibitory protein CD59 [31, 32] has influenced on the lesion expansion in murine NMO models. In the present study, it is unique that we successfully induced extensive primary astrocytopathy only by a single intraperitoneal injection of a high-affinity anti-AQP4 mAb which had severe clinical exacerbation and typical NMO-like lesions extending longitudinally from the medulla oblongata to spinal cord.

The merits of the NMO model with MBP-EAE have already been reported [42]. Normal MBP-EAE takes a monophasic course without causing marked demyelination and axonal injury with full recovery. In addition, it is difficult to find infiltrations of neutrophil or eosinophils in Lewis rat EAE, induced by emulsion of guinea pig MBP and CFA without administrating pertussis toxin additionally [42-44]. In contrast, in the present model, the clinical severity and the lesion size of astrocytopathy is clearly dependent on the dose of the anti-AQP4 antibody and marked neutrophil infiltration and tissue vacuolation was only seen with high amounts of IgG (80 mg of hIgG_NMO_ or 0.1–1 mg of E5415A). Therefore, we consider that the marked infiltration of neutrophils in this model is not due to the characteristics of EAE but to the administration of a high dose of NMO-IgG and changes in the lesion milieu, and we speculate that neutrophil-mediated cytotoxicity against astrocytes is induced by the pathogenic IgG itself. After multivalent binding of C1q to the Fc portion of NMO-IgG, the latter could bind to the orthogonal arrays of particle (OAP), where the assembled AQP4 could strongly activate the complement cascade [45] through the classical pathway [46]. Such activation could induce the secretion of anaphylatoxins, such as C3a and C5a, as a powerful chemoattractant for neutrophils [47, 48] and macrophages [48, 49]. IL-8 is also released by macrophages [50], and actually, the C5b-9 and IL-8 levels are elevated in the CSF of NMO patients [11, 51]. These findings imply that the deposition of a large amount of NMO-IgG at the borders of lesions could trigger potent complement activation or microgliosis, resulting in mobilizing neutrophils to the leading edges of lesions.

The neutrophils were mainly localized at the leading edges of AQP4 loss, and more so in the gray matter than in the white matter. It is well known that protoplasmic astrocytes, mainly localized at the gray matter, have numerous foot processes that are positive for AQP4, as compared with fibrous astrocytes that are mainly localized at the white matter [52, 53]. In the border area, the expression of AQP4 was relatively high at the outside of the border areas because of the presence of reactive astrogliosis with strong expression of AQP4, probably inducing the deposition of NMO-IgG and IgG-related immune cells, such as neutrophils. It is noted that neutrophils, eosinophils, and macrophages are the main infiltrating cells found in the lesions of human NMO, while infiltrated T lymphocytes [18] and natural killer cells [54] are rare. In our model, there was no infiltration of natural killer cells or eosinophils, thus neutrophils may have contributed to ADCC and promoted lesion expansion by secreting several kinds of cytokines, proteinases, and oxygen free radicals by activation of NADPH oxidase and neutrophil extracellular traps. Furthermore, the dominance of neutrophil involvement in the pathogenesis of NMO is supported by another previous study that most neutrophils are degranulated in a mouse model [55]. Moreover, it has been reported that a severe case of NMO occurred soon after the mistaken administration of G-CSF, a stimulator of granulocytes [56]. In the mouse DI model, the neutrophilia stimulated by G-CSF causes enlargement of the brain lesions; in contrast, the neutropenia induced by anti-neutrophil IgG reduced the size of these lesions [56]. In addition, a neutrophil protease inhibitor reduced loss of AQP4 in in vivo and ex vivo mouse models in the acute phase [56]. Therefore, in the present study, neutrophils were probably associated with development of NMO-like lesions via ADCC- and CDC-targeting astrocytes, and may represent a promising therapeutic target for antibody blocking reagents or blockers of neutrophil activation such as sivelestat sodium hydrate.

In our study, marked tissue vacuolation was seen at the lesion core in vasculocentric pathology, in contrast infiltrating neutrophils were found at the periphery of lesions in outward-spreading lesions. Like the localization of macrophage at the periphery in slowly expanding demyelinating lesions in MS [57, 58], the localization of neutrophil infiltration in the present study suggested that the neutrophil probably preceded tissue vacuolation in this model. In these lesions, loss of AQP4 and GFAP was observed along with the disintegration of perivascular GFAP-positive foot processes, and thus such tissue vacuolation may be derived from the lysis of perivascular astrocytes per se or the dysfunction of foot processes, inhibiting water circulation or absorption from the lesion to the subarachnoid space. Another possibility is intra-cellular edema; in the present study, some vacuolations were surrounded by MBP-stained fine structures, suggesting intra-myelin edema. Such tissue vacuolation has been reported in a double-knockout model of connexin 47 and connexin 30 [59]. Regardless, the tissue change is derived from astrocytopathy and may be specific to human NMO and rodent NMO models, in which some tissue vacuolations may develop into cavities as seen in autopsied cases of human NMO.

Epitopes of NMO-IgG that effectively induce clinical manifestation of the disease are unknown. NMO-IgG recognizes conformational epitopes of extracellular AQP4 loops and the lesions are never observed when recombinant unfolded protein is used as antigen in immunization [5]. Baculovirus display technology is a recently established method for generating mAbs to membrane proteins, preserving the conformational structure. E5415A made here using the baculovirus display method is an mAb against the extracellular domain of the M23 isoform of mouse AQP4, and could strongly bind to the rat extracellular AQP4 domains, with more than 256 − fold higher affinity as compared with the same dose of D15107 (supplement 3). In fact, in NMO/EAE model, we confirmed 1 mg E5415A model showed larger lesions than the same dose of D15107 model (data not shown). Therefore, strong binding properties with this mAb probably contributed to the strongly representative models of NMO produced here. However, further examination about E5415A will be needed to explain sufficiently why our model can reproduce such severe, extensive NMO-like lesions. There are some limitations in the present study. For instance, some differences in the amino acid sequences of extracellular AQP4 loops among human, rat, and mouse may influence the phenotype seen in our NMO model. It is still unknown why the optic lesions are relatively mild even in in vivo injection model with high amounts of pathogenic IgG in the present study, which needs further strict studies. Furthermore, we have observed the trend of typical AQP4-lacked lesions in gray matter and corticomedullary junctions with dominant infiltration of neutrophils likely independent from white matter perivascular cuffings, probably suggesting the involvement of neutrophils in addition to pioneering T cells for lesion expansion, but needs further detailed studies for understanding the pathomechanisms of NMO.

## Conclusion

In the present study, we established a severe and acute experimental NMO rat model clinically and pathologically extremely close to human NMO in the point of lesion size, clinical exacerbation course, and lesion localization, by high-affinity IgG against AQP4 made by baculovirus display method. Our data suggest that the pathogenic antibodies can induce the typical astrocytopathy with loss of AQP4 and GFAP, and mobilize neutrophils especially at AQP4 abundant lesion edge in dose dependent manner, resulting in early lesion expansion of NMO lesion with tissue vacuolation, secondary demyelination and axonal injury. Our model is likely to be useful in evaluating candidate drugs for NMO as well as in studying the pathomechanism of NMO.

## Ethical approval

All procedures performed in studies involving human participants were in accordance with the ethical standards of the institutional and/or national research committee and with the 1964 Helsinki declaration and its later amendments or comparable ethical standards. All procedures performed in studies involving animals were in accordance with the ethical standards of the institution or practice at which the studies were conducted (No.2015MdA-146).

## Availability of supporting data

The data sets supporting the results of this article are included within the article and its additional files.

## Additional files

Additional file 1: Table S1. Characteristics of in-vivo experimental NMO models (PDF 115 kb) Additional file 2:
**Cell-based affinity assay to assess binding of NMO-IgG to rat AQP4.** The comparison of each IgG binding affinity for rat AQP4-M23 in double dilution method. IgG were sprinkled on CHO cells expressing rat AQP4-M23 (Q222), and Alexa Fluor 568 or 594 was used as secondary antibody. This figure show enhanced green fluorescence protein (EGFP) and rat AQP4 staining when each IgG was diluted twice one after another from 1mg/ml (1/2^0^) to about 1000-fold dilution (1/2^10^), one million-fold (1/2^20^), and one billion-fold (1/2^30^). Rat AQP4 was not detected in hIgG_cont_ injected group, even in no dilution. In contrast, rat AQP4 was stained with cell membrane pattern, thought as IgG binding to rat AQP4. However, the difference of the binding was remarkable of the three groups. Rat AQP4 wasn’t detected at the timing of 2^17^ dilution in hIgG_NMO_, of 2^23^ dilution in D15107, but was detected even in about one billion dilution in E5415A. These findings show E5415A is the highest affinity monoclonal antibody for rat AQP4 among these three NMO-IgG used in the present study. (PDF 4513 kb) Additional file 3:
**Hemodynamics of E5415A injected intraperitoneally.** We gathered rat blood at the time of 0, 12, 24, 36 h (horizontal axis) from hIgGcont and E5415A (0.01 mg, 0.1 mg, 1 mg) injection. Baculovirus expressing mice AQP4-M1 isoform was immobilized in 96-well plate (2.5μg/well), where 100-fold diluted each serum was sprinkled. Next, 16,000-fold diluted goat anti-mouse IgG labeled by fluorescent molecule (A8924, Sigma-Aldrich, St. Louis, MO, USA) was used as secondary antibody, and absorption value (VA, vertical axis) was examined as the AQP4 antibody titration in sera. As a result, there is no elevation of AV in EAE (*n* = 1) and hIgGNMO groups (*n* = 2) without E5415A injection. In contrast, there is a dose-dependent increase of AV in E5415A groups. Every dose of E5415A groups showed maximum AV at 12 h after the injection, and peaked out in 0,01 mg and 0.1 mg E5415A groups. These findings suggest that E5415A injected intraperitoneally reach maximum blood concentration within 12 h in the context of EAE. (PDF 1251 kb)
