# Supplementary material for: Severely exacerbated neuromyelitis optica rat model with extensive astrocytopathy by high affinity anti-aquaporin-4 monoclonal antibody
Source: Acta Neuropathol Commun. 2015 Dec 4;3:82. doi: 10.1186/s40478-015-0259-2 (PMC4670539; doi:10.1186/s40478-015-0259-2)
Supplement: Additional file 1: Table S1. — Characteristics of in-vivo experimental NMO models (PDF 115 kb) [file 40478_2015_259_MOESM1_ESM.pdf]

Table 1. Characteristics of in-vivo experimental NMO models (2009-2015)

Reference	Model Type	Animal	IgG	Complement	IgG Injection Time	AQP4 Loss	GFAP Loss	Demyelination	Neutrophils	Lesion Localization	Clinical exacerbation
Present data ($hIgG_{NMO}$)	Passive IgG transfer model with EAE (MBP + CFA)	Lewis rat	IgG purified from sera of seropositive NMO patient, 20mg or 40mg, i.p.	(-)	48hrs after the onset	(+)	(+)	N.D	N.D	sp, chi, br	No (tail or hindlimb paresis in most EAE ctrls to sometimes forelimb paresis)
Present data (E5415A)	Passive IgG transfer model with EAE (MBP + CFA)	Lewis rat	Mouse anti-mouse mAb with high affinity for AQP4 (E5415A), 100μg or 1mg, i.p	(-)	48hrs after the onset	(+++)	(+++)	(±)	(+++)	sp, bs, chi, br	YES (tail or hindlimb paresis in most EAE-ctrls to forelimb paralysis or moribound)
Kinoshita, et al. 2009	Passive transfer model with EAE (MBP + CFA +PT)	Lewis rat	Pooled IgGs purified from IAPP column with 3 seropositive NMO patients, 20mg, 4 consequtive days, i.p.	(-)	24hrs after the last IgG injection	(++)	(++)	N.D	(+)	sp	Yes (tail or hindlimb paresis)
Bradl, et al. 2009	Active immunization model with EAE (MBP reactive T cell line)	Lewis rat	Pooled IgGs purified from sera or plasma exchange material of 6 patients with AQP-4 antibody–positive NMO or transverse myelitis, 10mg, 2 consequtive days, i.p.	(-)	24hrs after the last IgG injection	(++)	(++)	N.D	(++)	sp,bs, chi	YES (hindlimb paresis in ctrls to hindlimb plegia)
Bennett, et al. 2009	Passive transfer model with EAE (MBP + CFA)	Lewis rat	Four NMO CSF rAbs (rAb-10, rAb-168, rAb-51, rAb-43), a monoclonal antibody against MOG (mAb 8-18C5), and rAb-2B4, a control rAb against measles virus nucleocapsid, were transfused by retrobulbar venous plexus injection	(-)	30hrs after the IgG injection		(+++)	(±)		sp	N.D.

Kinoshita, et al. 2010	Passive transfer model with CFA only	Lewis rat	Pooled IgGs purified from IAPP column with 3 seropositive NMO patients, 20mg, 4 consequtive days, i.p.	(-)	24 hrs after the last IgG injection	(+)	(+)	N.D	(+)	sp	N.D.
Saadoun, et al. 2010	Intraventricular or intracerebral injection model	CD1 mice	Pooled IgGs purfied from sera of 5 patient with AQP-4 antibody–positive NMO, 3 times, intracerebral injection	hC or mC	(12 hrs or) 7 days after the last injection	Yes	Yes	Yes	(+)	br	N.D
Saadoun, et al. 2011	Intracerebral injection model	CD 1 mice vs nude mice	Pooled IgGs purfied from sera of 5 patient with AQP4 antibody–positive NMO, 3 times, intracerebral injection	hC	(24 hrs or) 5 days after the last injection	Yes		Yes	(+)	br	N.D
Pohl, et al. 2011	Active immunization model with EAE (AQP4-specific T cell line to AQP4$_{207–}$	Lewis rat	Pooled IgGs purified from sera or plasma exchange material of 6 patients with AQP4 antibody–positive NMO or transverse myelitis, 10mg, 2 consequtive days, i.p.	(-)	24 hrs after the injection	(++)				sp	N.D
Kitic, et al. 2013	Cytokine injection model (IL-1β, TNF-α, IL-6, IFN-γ, CXCL-2 and	Lewis rat	Pooled IgGs purified from sera or plasma exchange material of patients with AQP-4 antibody–positive NMO, 10mg, 2 consequtive days, i.p.	(-)	18-24hrs after the injection	(+)		N.D	(+)	br	N.D
Asavapanumas, et al. 2014	Intracerebral injection model	Lewis rat	Human recombinant monoclonal NMO antibody (rAb-53), 10μg	(-)	5 days after the injection	Yes	Yes	Yes	(±)	br	N.D
Asavapanumas, et al. 2014	Continuous perichiasmal infusion model	CD1 mice	Human recombinant monoclonal NMO antibody (rAb-53), 5μg, 3days continuous perichiasmal infusion	hC	The day of last injection	Yes	Yes	Yes	(+)	chi	N.D

Zhang, et al. 2014	Intrathecal injection	BALB/c wild-type vs CD59	Human recombinant monoclonal NMO antibody (rAb-53), 10μg, i.th.	hC	2 days after the injection	Yes	Yes	Yes	N.D	sp (LETM in white matter)	YES (almost intact in ctrls, to hindlimb paresis)
Wrzos, et al. 2014	Intracerebral injection	Lewis rat	1 μl of NMO rAb, NMO rAb_no ADCC (n = 8) or NMO rAb_no CDC (n = 8) (c = 2.5 mg/ml) diluted in NMO-IgG negative human serum or PBS	human serum or hC	various time point (1/3/24h, 3 days, 1/2/4 weeks)	Yes	Yes	Yes	Yes	br	N.D
Geis, et al. 2015	Intrathecal injection model	Lewis rat	(i)Pooled IgGs purified from plasma exchange material of 4 patients with AQP4 antibody-positive NMO, (ii) Human recombinant IgG Abs (Bennet, et al. 2009), 3 series of 5 daily applications with a 2-day interval between the 3 series, i.th.	(-)	1 to 3 days after the last injection	Yes	(±)	N.D	N.D	sp (adjacent to peridural catheter)	YES (tail hypotonus in most ctrls, to tail paralysis, hindlimb paresis)

hC: human complement, mC: mouse complement, N.D: not detected, sp: spinal cord, bs: brain stem, chi: optic chiasma

AQP4 loss/GFAP loss/Demyelination

(±)	not intact
(+)	loss area is localized at the glia limitance zround inflamed vessels.
(++)	loss area is generally limited in multiple monovessels, not fusional
(+++)	loss area is generally fused over the multi-vessels

Neutrophils

(±)	not intact
(+)	<500mm^2 or a few showed only high power field in figure
(++)	$<$ 1,000 mm^2
(+++)	$\geqq$ 1,000 mm^2
