# Supplementary figures and images for: Severely exacerbated neuromyelitis optica rat model with extensive astrocytopathy by high affinity anti-aquaporin-4 monoclonal antibody
Source: Acta Neuropathol Commun. 2015 Dec 4;3:82. doi: 10.1186/s40478-015-0259-2 (PMC4670539; doi:10.1186/s40478-015-0259-2)

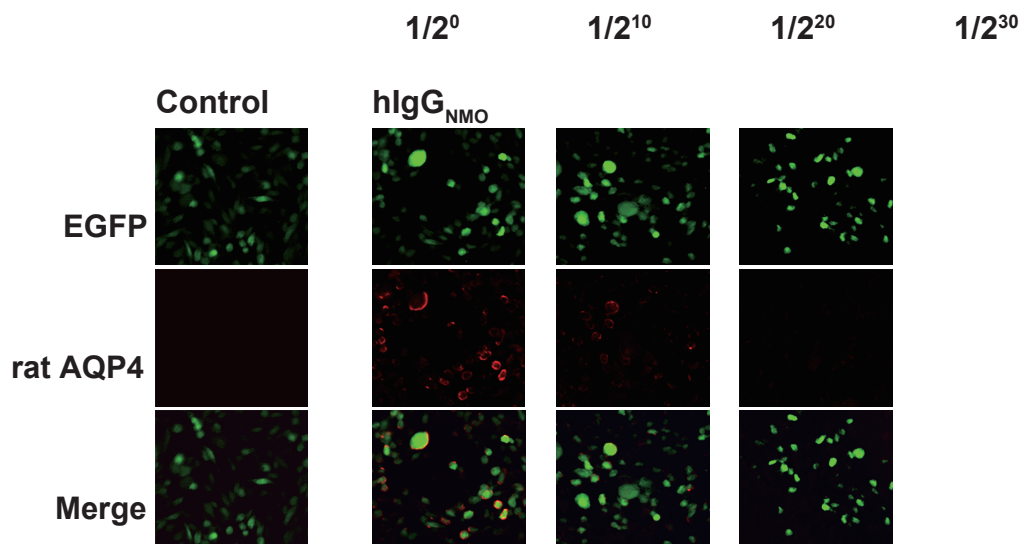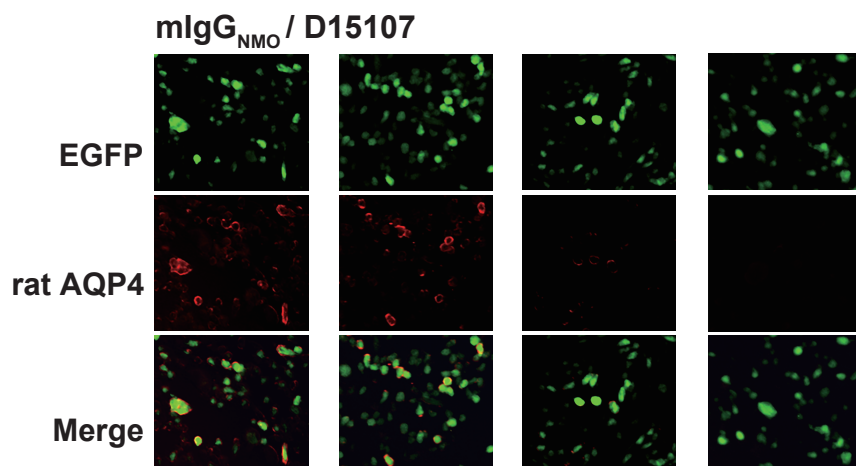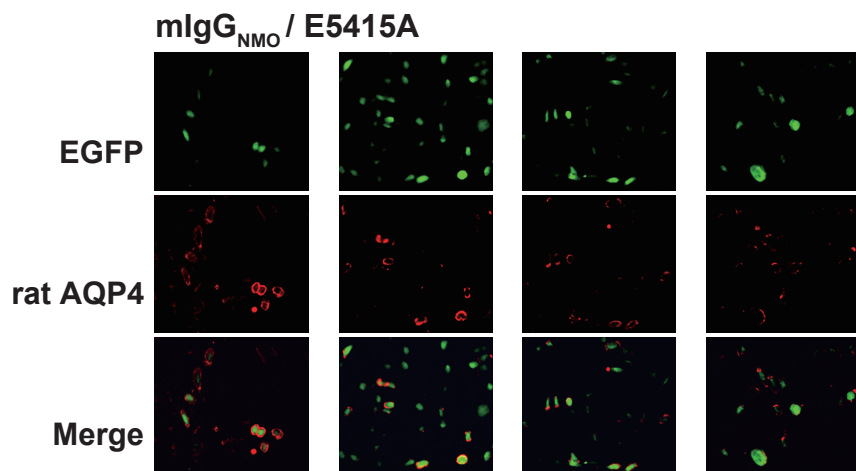

Supplement: Additional file 2: — Cell-based affinity assay to assess binding of NMO-IgG to rat AQP4. The comparison of each IgG binding affinity for rat AQP4-M23 in double dilution method. IgG were sprinkled on CHO cells expressing rat AQP4-M23 (Q222), and Alexa Fluor 568 or 594 was used as secondary antibody. This figure show enhanced green fluorescence protein (EGFP) and rat AQP4 staining when each IgG was diluted twice one after another from 1mg/ml (1/20) to about 1000-fold dilution (1/210), one million-fold (1/220), and one billion-fold (1/230). Rat AQP4 was not detected in hIgGcont injected group, even in no dilution. In contrast, rat AQP4 was stained with cell membrane pattern, thought as IgG binding to rat AQP4. However, the difference of the binding was remarkable of the three groups. Rat AQP4 wasn’t detected at the timing of 217 dilution in hIgGNMO, of 223 dilution in D15107, but was detected even in about one billion dilution in E5415A. These findings show E5415A is the highest affinity monoclonal antibody for rat AQP4 among these three NMO-IgG used in the present study. (PDF 4513 kb) [file 40478_2015_259_MOESM2_ESM.pdf]

[× 100 ng/ml]

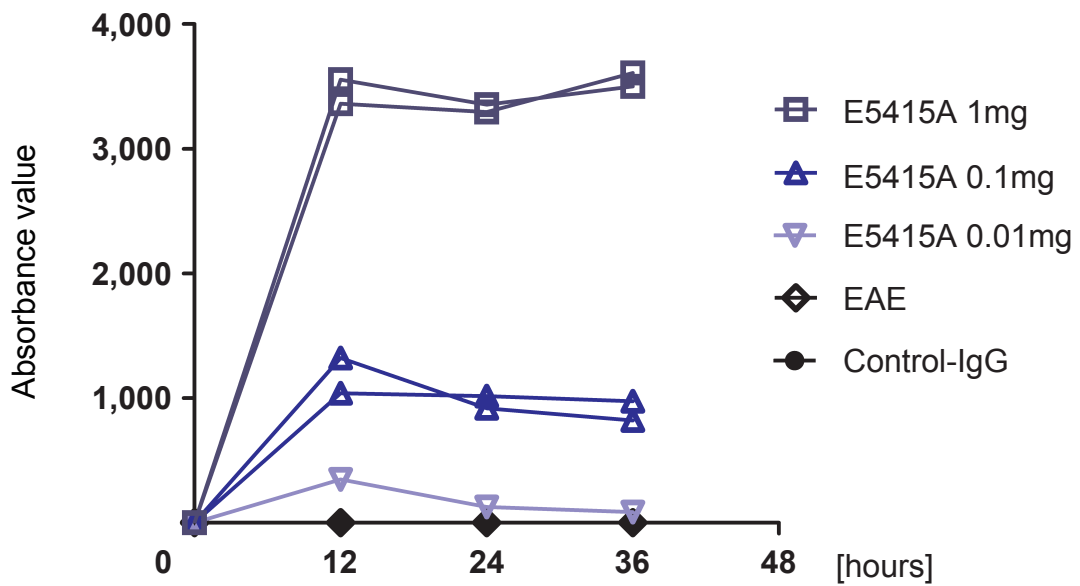

Supplement: Additional file 3: — Hemodynamics of E5415A injected intraperitoneally. We gathered rat blood at the time of 0, 12, 24, 36 h (horizontal axis) from hIgGcont and E5415A (0.01 mg, 0.1 mg, 1 mg) injection. Baculovirus expressing mice AQP4-M1 isoform was immobilized in 96-well plate (2.5μg/well), where 100-fold diluted each serum was sprinkled. Next, 16,000-fold diluted goat anti-mouse IgG labeled by fluorescent molecule (A8924, Sigma-Aldrich, St. Louis, MO, USA) was used as secondary antibody, and absorption value (VA, vertical axis) was examined as the AQP4 antibody titration in sera. As a result, there is no elevation of AV in EAE (n = 1) and hIgGNMO groups (n = 2) without E5415A injection. In contrast, there is a dose-dependent increase of AV in E5415A groups. Every dose of E5415A groups showed maximum AV at 12 h after the injection, and peaked out in 0,01 mg and 0.1 mg E5415A groups. These findings suggest that E5415A injected intraperitoneally reach maximum blood concentration within 12 h in the context of EAE. (PDF 1251 kb) [file 40478_2015_259_MOESM3_ESM.pdf]
